# Machine learning for neuroscience

**DOI:** 10.1186/2042-1001-1-12

**Published:** 2011-08-15

**Authors:** Geoffrey E Hinton

**Affiliations:** 1 Department of Computer Science, University of Toronto, Ontario, Canada

## What is machine learning?

Machine learning is a type of statistics that places particular emphasis on the use of advanced computational algorithms. As computers become more powerful, and modern experimental methods in areas such as imaging generate vast bodies of data, machine learning is becoming ever more important for extracting reliable and meaningful relationships and for making accurate predictions. Key strands of modern machine learning grew out of attempts to understand how large numbers of interconnected, more or less neuron-like elements could learn to achieve behaviourally meaningful computations and to extract useful features from images or sound waves.

By the 1990s, key approaches had converged on an elegant framework called 'graphical models', explained in Koller and Friedman, in which the nodes of a graph represent variables such as edges and corners in an image, or phonemes and words in speech. The probabilistic relationships between nodes are represented by conditional probability tables or simple functions whose parameters are learned from the data.

There are three main problems in fitting graphical models to data: inference, parameter learning and structure learning. The inference problem is how to infer the probable values of unobserved variables when the values of a subset of the variables have been observed, and is a problem that perceptual systems need to solve if they are to infer the hidden causes of their sensory input. The parameter-learning problem is how to adjust the parameters governing the way in which one variable influences another, so that the graphical model is a better fit to some observed data. In the brain, this is presumably done by changing synapse strengths. The structure-learning problem is how to decide which unobserved variables are needed and how they must be connected to model the correlations between observed variables. In the brain, evolution and early pruning of connections presumably have a large role to play in determining the structure.

## Could you provide a brief description of the methods of machine learning?

Machine learning can be divided into three parts: 1) in *supervised* learning, the aim is to predict a class label or a real value from an input (classifying objects in images or predicting the future value of a stock are examples of this type of learning); 2) in *unsupervised* learning, the aim is to discover good features for representing the input data; and 3) in *reinforcement* learning, the aim is to discover what action should be performed next in order to maximize the eventual payoff.

## What does machine learning have to do with neuroscience?

Machine learning has two very different relationships to neuroscience. As with any other science, modern machine-learning methods can be very helpful in analysing the data. Some examples of this include sorting the spikes picked up by an extracellular electrode into spike trains from different neurons, or trying to predict what object a person is thinking about by combining evidence from different voxels in a functional magnetic resonance imaging (fMRI) scan.

A much more interesting relationship is the use of machine-learning algorithms as a source of theories about how the brain works (and vice versa). For example, a currently influential theory of the functional role of an increase in dopamine level was inspired by an algorithm called td-learning, which was good at learning to select actions in games such as backgammon, in which the future contains uncertainty. Neuroscientists who are interested in learning ought to be aware of some of the more abstract principles that have already emerged from the field of machine learning. For example, methods for discovering the hidden causes of the sensory input can be divided into two broad classes, termed 'directed models' and 'undirected models'. To learn an undirected model in a neural network, it is necessary to include a phase in which the input is ignored and fantasy data are generated from the model. As was pointed out by Crick and Mitchison many years ago, this suggests a computational role for rapid eye movement (REM) sleep.

Most existing machine-learning methods for both *supervised* and *unsupervised* learning are shallow; they do not create multiple layers of adaptive features and so they are of limited interest to neuroscientists trying to understand perceptual pathways. The first widely used method for creating multiple layers of features was the *supervised* back-propagation algorithm, which was widely used in the 1980s, especially by psychologists. Unfortunately, it was difficult to see how it could be implemented in cortex, and its practical performance was somewhat disappointing because it required massive amounts of labeled data. For practical applications, it was largely replaced by a very clever shallow method called 'support vector machines'. More recently, however, we have discovered several *unsupervised* methods for creating multiple layers of features. one layer at a time. without requiring any labels. These methods are significantly better than the back-propagation method at creating useful high-level features, so 'deep' learning is making a comeback for tasks such as object and speech recognition. This should significantly enrich the interaction between machine learning and neuroscience.

## Will the machine-learning community build a machine smarter than we are, before or after we figure out how the brain works?

I think it is possible that a really smart machine will have to be massively parallel and will have to learn almost everything it knows in order to use its parallel hardware efficiently. If so, it may end up having to use learning procedures similar to those used in the brain, so building a really smart machine and understanding how the brain works may become the same project.

At present, the US Defense Advanced Research Projects Agency and the European Union seem determined to build the special-purpose hardware even before they know how to make it learn effectively, and I think that this is a massive mistake.

## Where can I find out more?

There are several good textbooks on machine learning. David MacKay explains machine learning from an information-theory perspective; Chris Bishop explains it from a strictly Bayesian perspective; Jerry Friedman, Trevor Hastie and and Rob Tibshirani explain it from a more eclectic statistics perspective; and Simon Haykin explains it from a signal-processing perspective. The book *Theoretical Neuroscience*, by Peter Dayan and Larry Abbott, has some nice examples of machine-learning ideas being applied to neuroscience. Further references are given in the bibliography.

## Bibliography

1. Probabilistic Graphical Models: Principles and Techniques (Adaptive Computation and Machine Learning), Daphne Koller and Nir Friedman, MIT Press, (2009), ISBN-10: 0262013193 ISBN-13: 978-0262013192

2. Information Theory, Inference and Learning Alogorithms, David J.C. MacKay, Cambridge University Press (2007), ISBN-10: 0521642981, ISBN-13: 978-0521642989

3. Pattern Recognition & Machine Learning, Christopher M. Bishop, Springer (2007), ISBN-10: 0387310738, ISBN-13: 978-0387310732

4. The Elements of Statistical Learning: Data Mining, Inference, and Prediction. Trevor Hastie, Robert Tibshirani and Jerome Friedman. Springer (2009) ISBN-10: 0387848576, ISBN-13: 978-0387848570

5. Neural Networks and Learning Machines. Simon Haykin. Prentice Hall (2009), ISBN-10: 0131471392, ISBN-13: 978-0131471399

6. Theoretical Neuroscience: Computational & Mathematical modelling of neural systems. Peter Dayan and Larry Abbott, MIT Press (2005), ISBN-10: 0262541858, ISBN-13: 978-0262541855

7. Crick and Mitchison: The function of dream sleep. *Nature *1983, 304: 111-114

